# Fabrication of an α-Fe_2_O_3_ NP-modified ZnO NRs/Ni-foam nanocomposite electrode for electrochemical detection of arsenic in drinking water

**DOI:** 10.1039/d4ra07509a

**Published:** 2024-11-26

**Authors:** Sreymean Ngok, Rem Yann, Chan Oeurn Chey, Xianjie Liu, Magnus Willander, Omer Nur

**Affiliations:** a Department of Science and Technology, Physics Electronics and Mathematics, Linköping University SE-60174 Norrköping Sweden sreymean.ngok@liu.se +46 11 36 32 19; b Laboratory of Organic Electronics, ITN, Linköping University Norrköping SE-60174 Sweden; c Graduate School of Science, Royal University of Phnom Penh Phnom Penh Cambodia; d Department of Physics, Faculty of Science, Royal University of Phnom Penh Phnom Penh Cambodia

## Abstract

Arsenic is a toxic contaminant that can be found in drinking water. In this study, the development of an efficient electrode as an electrochemical sensor to detect arsenic(v) in drinking water is presented. The surface of ZnO nanorods (NRs) synthesized on a Ni-foam substrate was modified by depositing α-Fe_2_O_3_ nanoparticles (NPs) to fabricate an electrode for the detection of arsenic(v) contamination in drinking water. This electrode was synthesized through two separate growth steps: a hydrothermal (ZnO NRs) step followed by the dip-coating method (α-Fe_2_O_3_ NPs). The dip-coating method was repeated multiple times, 2 times (ZNF-2), 3 times (ZNF-3) and 4 times (ZNF-4), in order to achieve a uniform coverage of the ZnO NR surface. The electrodes were characterized using XRD, XPS, SEM and UV-vis spectroscopy. The best efficiency among the α-Fe_2_O_3_NP-modified nanorod samples was observed for the 3-time dip-coated ZNF-3 sample, which presented a uniform and homogeneous morphology, as observed from the SEM images, accompanied with the highest oxidation current. The electrochemical performance of the sensor electrodes was tested for a wide range of arsenic(v) concentrations from 0 to 50 ppb and was monitored using cyclic voltammetry. The results demonstrated a calibration plot that was linear over a concentration range of 0–50 ppb of arsenic(v), and the regression equation extracted from the calibration curve was found to be *y* = 0.003*x* − 0.6271 (with *R*^2^ = 0.991). The limit of detection (LOD) and limit of quantification (LOQ) were found to be 4.12 ppb and 13.74 ppb, respectively, which are lower than the maximum allowed value recommended by the World Health Organization (WHO) for arsenic in drinking water. This reasonable performance of the ZnO NRs/Ni-foam/α-Fe_2_O_3_NP nanocomposite electrode can be further enhanced, and the electrode can be utilized for efficient arsenic(v) detection in drinking water.

## Abstract 2

Arsenic is a toxic contaminant that can be found in drinking water.

## Graphical abstract

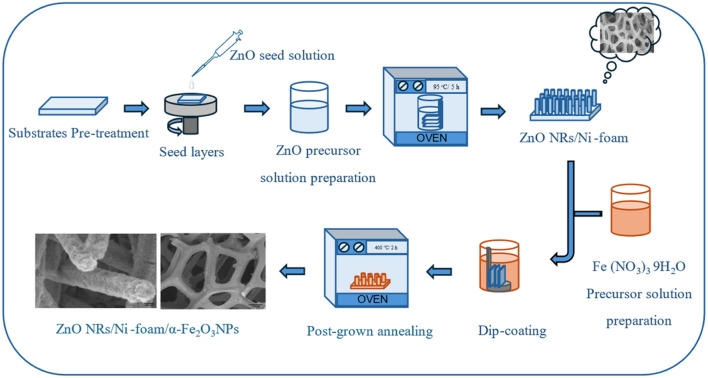

## 1 Introduction

Arsenic is known as a naturally occurring toxic element and is considered one of the world's environment hazardous agents.^1^ Arsenic is generally found in two forms, arsenite (As(iii)) and arsenate (As(v)), which are among the inorganic forms of arsenic and the predominant toxic species found in natural drinking water.^2^ The World Health Organization has fixed the maximum contaminant level of arsenic in drinking water at 50–10 ppb.^3–5^ Therefore, the detection of the trace amount of arsenic has received considerable attention.

Metal oxide nanomaterials have more sensing ability than single-crystalline bulk semiconductor metal oxide.^6^ Furthermore, they exhibit many attractive features such as high chemical and thermal stability, high surface area to volume ratio, tunable electronic states, quantum confinement, high electron mobility, and excellent catalytic properties.^7^ Various types of metal oxide nanomaterials have been explored for sensing applications, *e.g.* tin oxide (SnO_2_), indium oxide (In_2_O_3_), zinc oxide (ZnO), tungsten trioxide (WO_3_), cuprous oxide (Cu_2_O), cobalt oxide (Co_3_O_4_) and hematite (α-Fe_2_O_3_).^8,9^ ZnO and α-Fe_2_O_3_ are interesting oxides that are abundant, can be achieved using low cost synthesis methods and are considered less toxic materials.^10,11^ Further, different nanocomposites of these oxides with promising synergistic advantageous properties have been reported. Among these nanocomposite materials, ZnO nanostructures modified with α-Fe_2_O_3_ nanostructures have been effective.^12–16^ The composition of α-Fe_2_O_3_ and ZnO nanostructures results in their synergistic effect, which can enhance the performance of the composite nanomaterial.^7,17–20^ These nanocomposites have gained interest in the field of sensor development due to their small dimensions, simplicity of the synthesis procedure, and the possibility of fast sensor response.^21–24^ However, these nanomaterials can have some disadvantages, such as low sensitivity, poor selectivity and high operating temperatures, which discourage their utilization in different fields.^24,25^ Thus, many researchers have made efforts to improve the sensitivity and selectivity of these metal oxides by compositing them with other metal oxide semiconductors.^9,26–31^ In this regard, we have fabricated α-Fe_2_O_3_ nanoparticles on the surface of ZnO NRs grown on a nickel foam substrate in this work for the detection of arsenic(v) in drinking water. The nickel foam substrate has loose porosity, good stability and uniform heat transfer.^32^ In addition, nickel foam is a commercial material with a 3D porous structure and can be utilized as an efficient electrode/substrate material as it provides excellent electrical conductivity and a large enough surface area, enabling good access for ions and electrons to reach the active surface of the hybrid composite electrode.^33,34^ Moreover, Nickel foam substrates can be used not only to enhance the conductivity performance and promote the penetration of electrolytes in practical applications, but nickel foam has also been used as a current collector in various electrochemical systems, including energy storage devices, chemical sensors and wastewater treatment systems.^35–37^

Herein, we fabricated α-Fe_2_O_3_NPs on ZnO NRs/Ni-foam substrate for efficient arsenic(v) detection in drinking water. The ZnO NRs/Ni-foam/α-Fe_2_O_3_NPs nanocomposite electrode was grown *via* two separate growth steps: hydrothermal reaction followed by a dip-coating process. The dip-coating step was optimized by carrying the dipping cycles. The structural and optical properties of the fabricated electrode were then characterized using different techniques, such as XRD, XPS, SEM and UV-vis spectroscopy. The electrochemical performance of the sensor electrode in arsenic(v) detection was investigated by cyclic voltammetry in various electrolytes to achieve efficient detection of arsenic(v). The performance of the ZnO NRs/Ni-foam/α-Fe_2_O_3_NP nanocomposite electrode in the detection of arsenic(v) in drinking water is promising, verifying it as an excellent candidate that can further be developed to enhance detection efficiency.

## 2 Experimental methods

### 2.1 Materials

Zinc nitrate hexahydrate (Zn(NO_3_)_2_·6H_2_O), hexamethylenetetramine (HMT), iron(iii) nitrate nonahydrate (Fe(NO_3_)_3_·9H_2_O) and arsenic standard solutions were purchased from Sigma Aldrich. Nickel foams (10 mm × 15 mm × 1.5 mm) were purchased from Redox.me (Sweden). All chemicals were of analytical grade and used without further purification.

### 2.2 Fabrication of ZnO NRs/Ni-foam and the ZnO NRs/Ni-foam/α-Fe_2_O_3_NPs nanocomposite

**Fig. 1:**
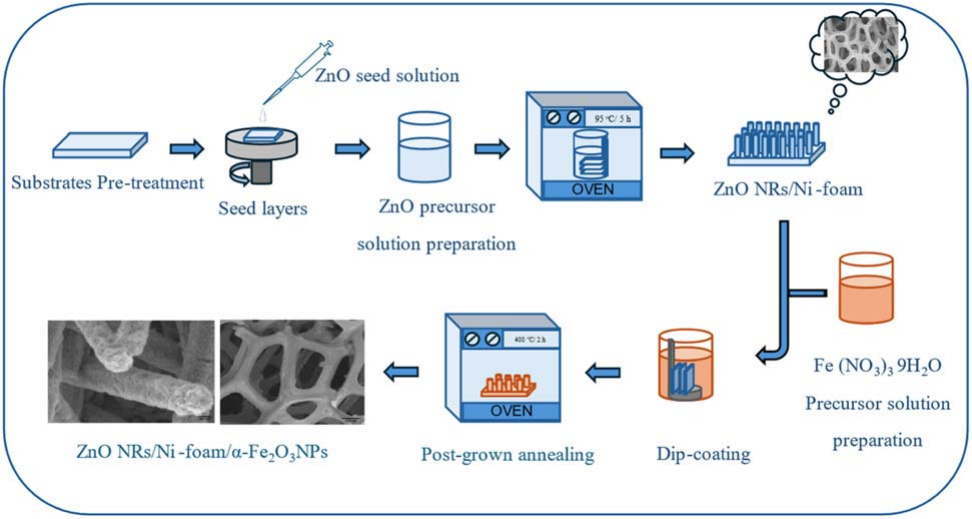
Schematic showing the process for synthesis of the ZnO NRs/Ni-foam/α-Fe_2_O_3_NPs nanocomposite electrode.

The electrode was fabricated in two steps. During the first step, ZnO NRs were grown *via* the hydrothermal method. Briefly, the nickel foam substrate was cut into small pieces (10 mm × 15 mm × 1.5 mm), cleaned sequentially with deionized water, acetone, and isopropanol in an ultrasonic bath for 10 minutes each and then dried under a nitrogen flow at room temperature. The cleaned substrates were dip-coated using a seed layer of ZnO nanoparticles for 5 minutes. The seed layer-coated substrates were annealed at 120 °C for 20 min. The seed solution was prepared by dissolving zinc acetate dihydrate (0.01 M) in methanol (125 mL) under vigorous stirring at a temperature of about 60 °C. Subsequently, a 0.03 M solution of KOH (65 mL) in methanol was added dropwise at 60 °C. The mixture was stirred for 2 h at 60 °C and then at room temperature for 12 h to obtain the desired rod-shaped particles.^38^ The precursor solution used to synthesize the ZnO NRs was an equal molar (0.05 M) solution of Zn(NO_3_)_2_·6H_2_O and HMT in 100 mL deionized water. Finally, the annealed nickel foam substrates were fixed to a sample holder with the ZnO-seeded sides pointing downwards and dipped into the ZnO precursor solution. After that, the beaker containing the precursor solution and the seeded samples was closed using aluminum foil and baked in an electronic oven at a temperature of 95 °C for 5 h. After the completion of the growth period, the samples were taken out from the oven, washed with deionized water, and then dried using a nitrogen flow. In the second step, α-Fe_2_O_3_ NPs were synthesized by the dip-coating method. The modification of ZnONRs/Ni-foam substrate with α-Fe_2_O_3_ NPs was achieved by dipping the as-grown ZnONRs/Ni-foam into a precursor solution of 0.06 g Fe(NO_3_)_3_·9H_2_O dissolved in 20 mL of deionized water. The products were dipped for 2 minutes per cycle; the dipping process was repeated two, three or four times, and the samples were denoted as (ZNF-2), (ZNF-3) and (ZNF-4), respectively. This was followed by drying under flowing nitrogen at room temperature. The final products were annealed at 400 °C for 2 h in an air environment to convert the hydroxyl-containing phase of Fe_2_O_3_ to pure α-phase (Fig. 1).

### 2.3 General characterization techniques

The morphology of the electrodes was characterized by field-emission scanning electron microscopy (FESEM, Sigma 500 Gemini) with the field-emission gun operated at 10 kV. The structure and phase of the grown nanocomposite were investigated by powder X-ray diffraction (XRD) using a Philips powder diffractometer equipped with Cu Kα radiation at a voltage of 45 kV and a current of 40 mA. The optical properties were analyzed using a UV-vis spectrophotometer (PerkinElmer Lambda 900). The oxidation states and chemical composition were analyzed by X-ray photoelectron spectroscopy (XPS).

### 2.4 Electrochemical characterizations

The electrochemical measurements were carried out using an Autolab potentiostat (Metrohm). The electrochemical properties were measured in a three-electrode configuration composed of Ag/AgCl (3 M KCl) as the reference electrode, platinum wire as the counter electrode and ZnO NRs/α-Fe_2_O_3_ nanoparticles as the working electrode. The geometric area of the working electrode was estimated to be 0.3 cm^2^. The supporting electrolyte was a 1 M KOH solution with arsenic. All measurements were made in a fume hood at room temperature.

## 3 Results and discussion

### 3.1 Morphological analysis

**Fig. 2:**
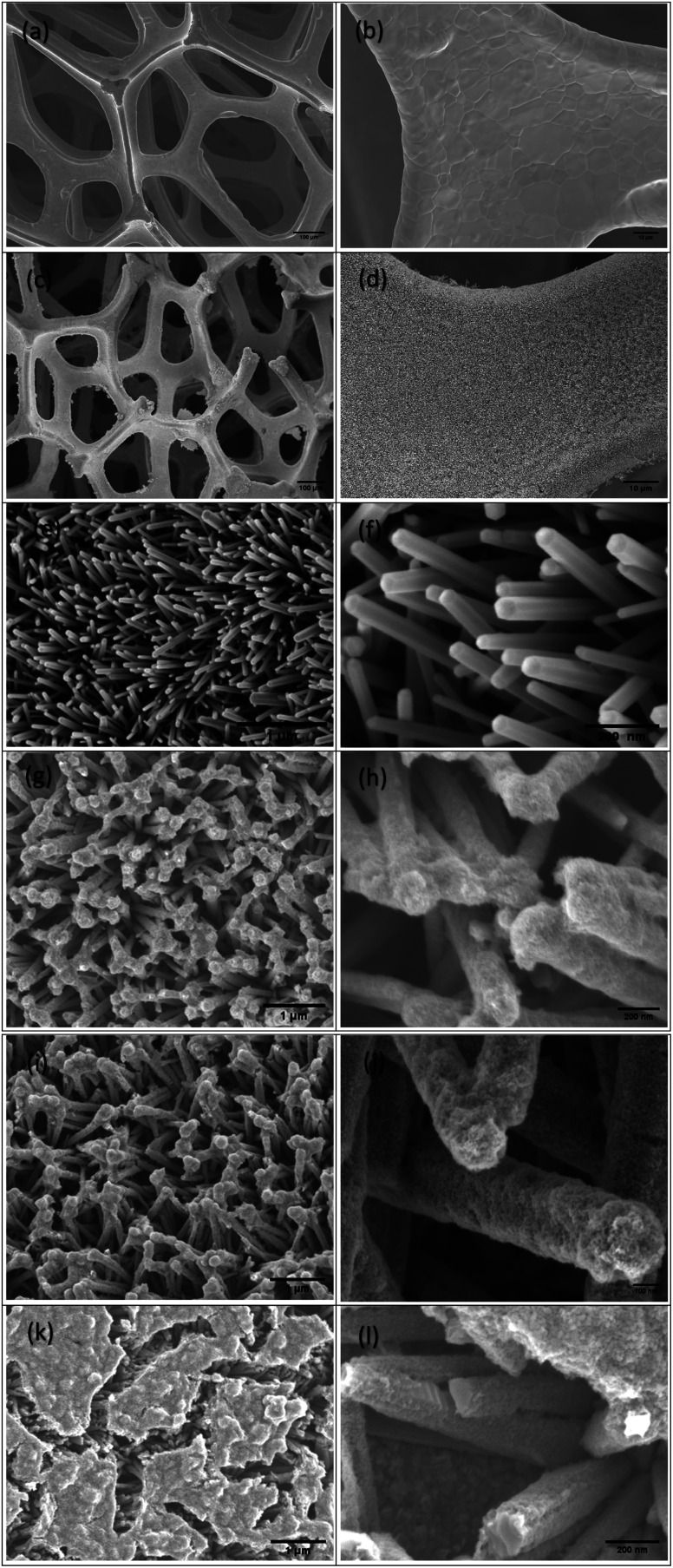
The SEM images of (a and b) the nickel foam substrate, (c–f) ZnO NRs/Ni-foam and ZnONRs/Ni-foam/α-Fe_2_O_3_NPs (ZNF-2) (g and h), (ZNF-3) (i and j) and (ZNF-4) (k and l).

The surface morphology of the ZnO NRs/Ni-foam/α-Fe_2_O_3_NPs nanocomposite was examined by FESEM. Fig. 2a and b show the FESEM images of the bare Ni-foam substrate, while (Fig. 2c–f) show the morphology after the growth of the nanorods. Fig. 2f shows a high-magnification FESEM image of the ZnO NRs grown on the nickel foam substrate, demonstrating the expected hexagonal shape. The ZnO NRs were dense highly uniform, and relatively vertically aligned. Fig. 2g–l demonstrate that the α-Fe_2_O_3_ NPs were deposited on the ZnO NRs. The surfaces of the ZnO NRs modified with α-Fe_2_O_3_ NPs using different cycles (2, 3 and 4) of dip coating are shown in Fig. 2(g, h), (i, j) and (k, l), respectively. The ZnO NRs surface immersed 2 times in the α-Fe_2_O_3_ NPs precursor solution for 2 minutes indicated partial covering of the ZnONRs surface with α-Fe_2_O_3_ nanoparticles (Fig. 2g and h). However, the ZnO NR surface immersed 3 times in the α-Fe_2_O_3_ NPs precursor solution for 2 minutes each showed full coverage, with gaps between the ZnO NRs (Fig. 2i and j). Furthermore, the gaps between the ZnO NRs were filled with α-Fe_2_O_3_ NPs after immersing the NRs 4 times in the precursor solution for 2 minutes each (Fig. 2k and l).

### 3.2 Optical properties

**Fig. 3:**
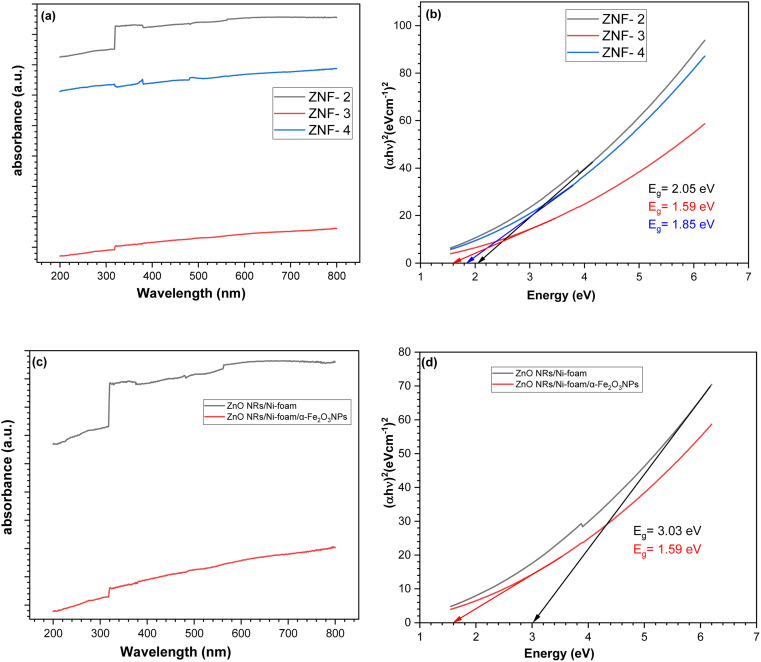
UV-vis absorption spectra of (a) the ZnO NRs/Ni-foam/α-Fe_2_O_3_NPs nanocomposite with different dip coatings. (b) Evaluation of bandgap energies of (ZNF-2), (ZNF-3) and (ZNF-4). (c and d) UV-vis absorption spectra and evaluation of bandgap energies of the bare ZnO NRs/Ni-foam and ZnO NRs/Ni-foam/α-Fe_2_O_3_NPs nanocomposite.

The optical characterization of the sample was carried out by UV-visible spectroscopy. In Fig. 3a and b, the absorption *versus* wavelength plots of the α-Fe_2_O_3_NP-coated samples obtained by varying the number of dip-coating cycles are displayed. The sample synthesized with 2, 3 and 4 dip-coating cycles of pure ZnO NRs/Ni-foam showed different reflectance values in the visible-light region. ZNF-2, ZNF-3 and ZNF-4 were found to have bandgap energies of approximately 2.05 eV, 1.59 eV and 1.85 eV, respectively, as shown in Fig. 3b. The sample dip-coated 3 times showed the highest absorption in the visible-light region. In Fig. 3c, bare ZnO NRs/Ni-foam shows strong absorption in the ultraviolet range, but there is also fluctuation in light absorption in the 200–400 nm region, with broadening of the optical band. However, the spectrum of ZnONRs/Ni-foam/α-Fe_2_O_3_NPs shows a characteristic absorption edge at 317 nm and a broader absorption range extending to the visible light region. With an increase in the α-Fe_2_O_3_ NPs layer thickness, there was an increase in the optical absorption coefficient of the ZnO NRs/Ni-foam/α-Fe_2_O_3_NPs samples at longer wavelengths compared to ZnO NRs/Ni-foam. ZnO NRs/Ni-foam and the ZnO NRs/Ni-foam/α-Fe_2_O_3_NPs nanocomposite revealed bandgap energies of approximately 3.03 eV and 1.59 eV, respectively, as shown in Fig. 3d.

### 3.3 XRD analysis

**Fig. 4:**
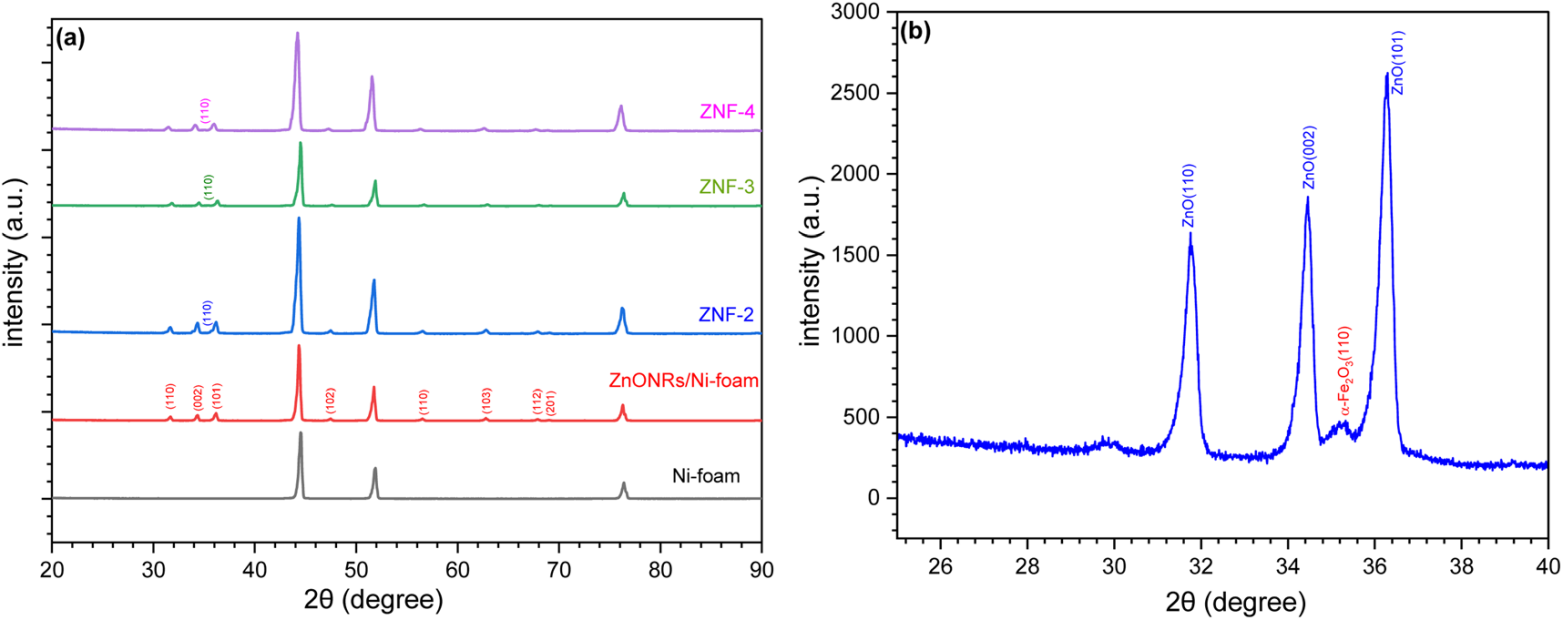
(a) XRD patterns of Ni-foam, ZnO NRs/Ni-foam, (ZNF-2), (ZNF-3) and (ZNF-4). (b) High-resolution XRD pattern of ZNF-3.

The crystallinity and phase purity of ZnONRs/Ni-foam and ZnONRs/Ni-foam/α-Fe_2_O_3_NPs were characterized by X-ray diffraction (XRD), as shown in Fig. 4a. The XRD pattern of the bare Ni-foam was recorded from 20° to 90°. The diffraction peaks of bare Ni-foam at 44.50°, 51.89° and 76.41° match well with the standard JCPDS card (JCPDS no.: 003-1051). The diffraction peaks of ZnONRs/Ni-foam at 2*θ* positions 31.66°, 34.40°, 36.15°, 47.50°, 56.58°, 62.78°, 67.93° and 69.02° attributed to the (110), (002), (101), (102), (110), (103), (112) and (201) planes, respectively, were due to the hexagonal wurtzite structure of the ZnO NRs grown on the nickel foam surface and matched JCPDS no. 00-005-0664 in the diffraction database. The additional diffraction peaks of the ZnO NRs/Ni-foam/α-Fe_2_O_3_NPs nanocomposite samples obtained 2 (ZNF-2), 3 (ZNF-3) and 4 cycles (ZNF-4) of dip coating were at 35.27°, corresponding to the (110) reflection planes of the rhombohedral structure of the α-Fe_2_O_3_ NPs, and matched the JCPDS: 00-033-0664 file from the diffraction database, as shown in Fig. 4b.

### 3.4 XPS analysis

**Fig. 5:**
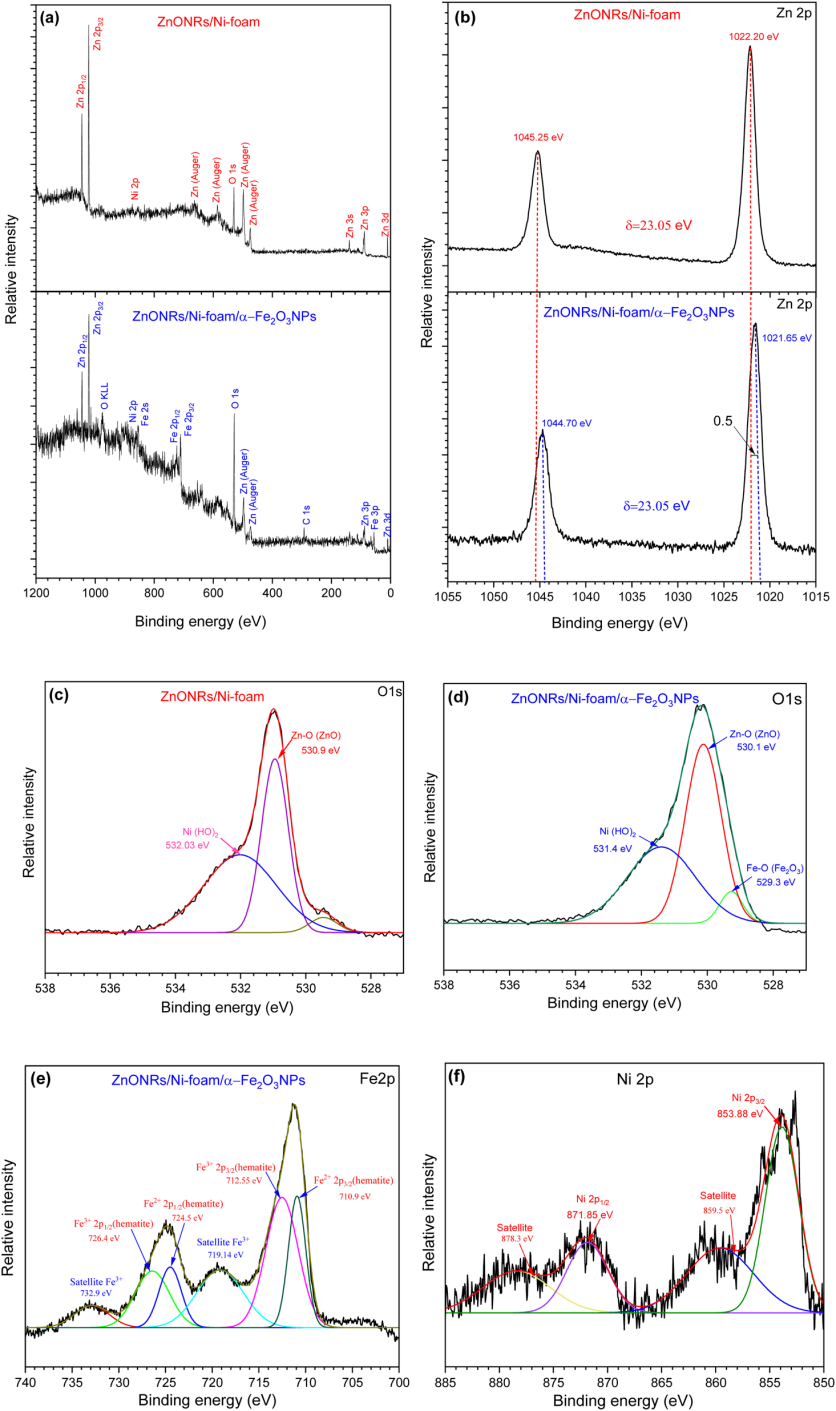
XPS analysis of the ZnO NRs/Ni-foam and ZnO NRs/Ni-foam/α-Fe_2_O_3_ NPs nanocomposite: (a) survey spectra; high-resolution (b) Zn 2p, (c and d) O 1s, and (e) Fe 2p spectra; (f) Ni 2p spectrum of the nickel foam substrate.

X-ray photoelectron spectroscopy (XPS) measurements were carried out to investigate the chemical bonding states and elemental composition of ZnO NRs/Ni-foam and the ZnO NRs/Ni-foam/α-Fe_2_O_3_ NPs nanocomposite samples; the results were fitted by a Gaussian fitting method and are shown in Fig. 5. In Fig. 5a, the survey spectrum of bare ZnO NRs/Ni-foam is shown. It confirms the presence of Zn 3d, 3p, 3s, 2p, auger, O 1s and Ni 2p. After the modification of the ZnONRs/Ni-foam surface by depositing α-Fe_2_O_3_ NPs, the survey spectrum included peaks of Fe 3p, 2p, 2s, 0 KLL, and C 1s.^39–42^ The high-resolution Zn 2p spectra of the samples are shown in Fig. 5b; doublet peaks are observed at binding energies of 1022.20 eV and 1045.25 eV corresponding to pure ZnONRs/Ni-foam. This is consistent with the oxidation states of Zn 2p_3/2_ and Zn 2p_1/2_, respectively. However, these oxidation states were shifted to binding energies of 1021.65 eV and 1044.70 eV, respectively, for the ZnO NRs/Ni-foam/α-Fe_2_O_3_ NPs nanocomposite sample. Moreover, a spin–orbit energy separation of 23.05 eV demonstrates that ZnO had the wurtzite crystal structure (hexagonal),^43,44^ which is also consistent with the XRD result. The O 1s core-level XPS spectra of pure ZnO NRs/Ni-foam and the ZnO NRs/Ni-foam/α-Fe_2_O_3_NPs nanocomposite samples shown in Fig. 5c and d exhibit peaks at binding energies 530.9 eV and 530.1 eV, corresponding to the bonding of ZnO in the ZnONRs/Ni-foam and ZnO NRs/Ni-foam/α-Fe_2_O_3_NPs nanocomposite samples, respectively.^45,46^ The negative shift of the Zn 2p (by −0.5 eV towards the lower binding energy region) and O 1s peaks indicates that a heterojunction was formed by interfacial interaction in the ZnONRs/Ni-foam/α-Fe_2_O_3_NP nanocomposite.^47–52^ The other fitted peaks of the high-resolution O 1s spectrum of the ZnO NRs/Ni-foam/α-Fe_2_O_3_NPs nanocomposite located at binding energies 529.3 eV and 531.4 eV could be attributed to Fe–O(Fe_2_O_3_) and Ni(H_2_O), as shown in Fig. 5d.^53^ Meanwhile, the peak at 531.4 eV is characteristic of H_2_O, which can be crystal water or water adsorbed on the samples from the air, and it shifted to 532.03 eV in the case of bare ZnO NRs/Ni-foam.^36^ The core-level Fe 2p spectra showed Fe^2+^ 2p_3/2_, Fe^3+^ 2p_3/2_, Fe^2+^ 2p_1/2_ and Fe^3+^ 2p_1/2_ peaks at 710.9 eV, 712.55 eV, 724.5 eV and 726.4 eV, respectively, as displayed in Fig. 5e, indicating the presence of Fe^2+^ and Fe^3+^ species.^40,54^ Additionally, two satellite peaks were observed at 719.14 eV and 732.9 eV.^55,56^Fig. 5f shows the Ni 2p_3/2_ and Ni 2p_1/2_ XPS peaks at 853.88 eV and 871.85 eV, corresponding to the metallic Ni foam substrate.^39,40,57,58^ Based on the XRD and XPS results, it was concluded that α-Fe_2_O_3_NPs-modified ZnO NRs were successfully grown on the nickel foam substrate.

### 3.5 Electrochemical behaviors

#### 3.5.1 Cyclic voltammetry analysis

**Fig. 6:**
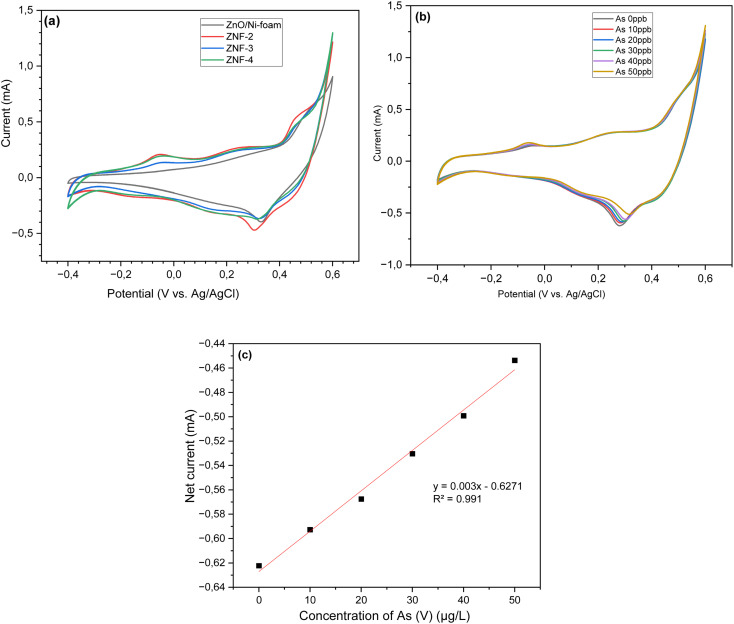
Cyclic voltammetry curves of (a) ZnONRs/Ni-foam, (ZNF-2), (ZNF-3) and (ZNF-4) electrodes in a 1 M KOH electrolyte (b). The ZNF-3 electrode at different concentrations of arsenic(v) in the potential range from −0.4 to 0.6 V at a scan rate of 100 mV s^−1^; (c) the corresponding linear calibration plot of net current against arsenic(v) concentration.

Cyclic voltammetry (CV) measurements of bare ZnO NRs/Ni-foam and the ZnO NRs/Ni-foam/α-Fe_2_O_3_ NPs nanocomposite were performed in 100 mL electrochemical cells to investigate the electrochemical behavior of the electrode material using 70 mL of a 1 M KOH solution and arsenic(v) at different concentrations. The CV curves were recorded in a potential window ranging from −0.4 to 0.6 V *vs.* Ag/AgCl at a scan rate of 100 mV s^−1^ in the 1 M KOH electrolyte. The oxidation and reduction reactions at the working electrode surface are shown by the CV profiles in Fig. 6a. The CV profiles of bare ZnO NRs/Ni-foam and the ZnO NRs/Ni-foam/α-Fe_2_O_3_NPs nanocomposites obtained after different dip-coating cycles showed that the oxidation and reduction peak currents were higher for the nanocomposite. The highest current was observed in ZnO NRs/Ni-foam/α-Fe_2_O_3_NPs nanocomposites, of which ZNF-2 showed a lower current than ZNF-3 and ZNF-4. However, ZNF-3 and ZNF-4 exhibited the same current. In Fig. 6b, the CV curves of the ZNF-3 electrode at different concentrations of arsenic(v) from 0–50 μg per L (ppb) demonstrate that the oxidation and reduction peak current increased with increasing arsenic(v) concentration in the electrolyte. The maximum oxidation and reduction currents 1.3 mA and −0.45 mA were achieved at voltage values of 0.6 V and 0.28 V, respectively, at 50 ppb arsenic(v) concentration. The calibration plot was found to be linear in a wide concentration range of 0–50 ppb of arsenic(v), and the regression equation extracted from the calibration curve was found to be *y* = 0.003*x* − 0.6271 (*R*^2^ = 0.991), as shown in Fig. 6c. These findings show the successful detection of arsenic(v) in an aqueous solution by the as-developed electrode. For further quantitative analysis, the limit of detection (LOD) and the limit of quantification (LOQ) of the sensor developed in this work were evaluated. Further, the LOD and LOQ results reported by others are shown in Table 1. The LOD is the minimum amount of analyte detected by the system, while LOQ is the lowest amount of analyte that can be quantitatively measured. The World Health Organization (WHO) has set 50 to 10 ppb as the maximum allowed arsenic content in drinking water.^4,5,64^ In this work, the LOD and LOQ of the developed ZnO NRs/Ni-foam/α-Fe_2_O_3_NPs nanocomposite electrode were calculated using the following equations:

1 LOD = 3*σ*/*S*

2 LOQ = 10*σ*/*S*

where *σ* is the standard deviation, and *S* is the slope of the calibration curve of current *vs.* concentration.^65^ From eqn (1) and (2), the calculated LOD and LOQ values of the ZnO NRs/Ni-foam/α-Fe_2_O_3_NPs nanocomposite electrodes were found to be 4.12 ppb and 13.74 ppb, respectively. Although these values are quite high compared to other methods, the electrode can only detect arsenic levels lower than the maximum value recommended by WHO. These CV measurements were repeated several times, and the ZnO NRs/Ni-foam/α-Fe_2_O_3_NPs nanocomposite sensor electrode was found to be relatively stable.

**Table 1:** Comparative study of the performance of different electrochemical sensors developed for arsenic detection

Materials	Detection method	Arsenic concentration	LOD	Ref.
Fe_3_O_4_ nanoparticles/nucleobase	SWV	1.1–0.5 μM	0.01 ppm	59
Fe_3_O_4_ nanocrystals modified GCE	SWASV	0.08–1.54 μM	0.01 ppm	60
Fe_3_O_4_ NPs		0–100 ppb	30 ppb	61
ZnO quantum dots		10–100 ppb	28 ppb	62
ZnO-NRs@Ni-foam	CV	0.1–1.0 μM	0.046 ppm	63
ZnO NRs/Ni-foam/α-Fe_2_O_3_NPs nanocomposite	CV	10–50 ppb	4.12 ppb	This work

#### 3.5.2 Interference effect

**Fig. 7:**
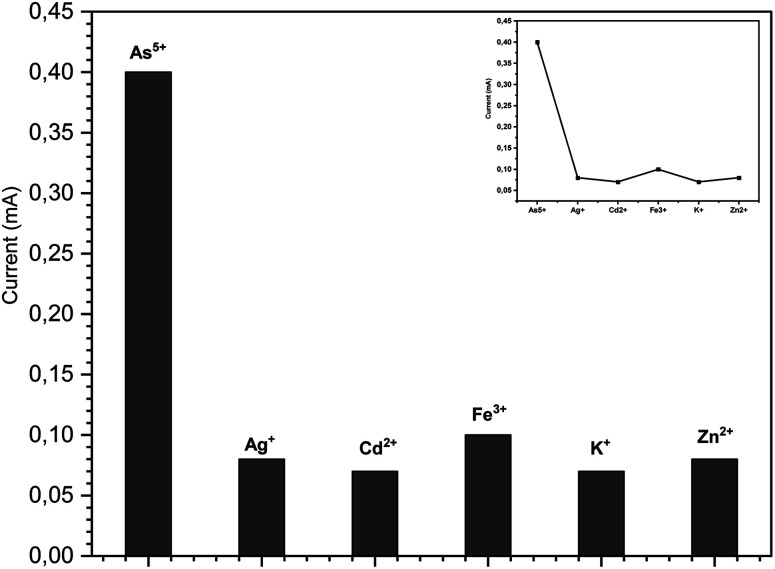
Interference effect of different metal ions under similar experiment conditions used for the detection of arsenic(v) by the ZnO NRs/Ni-foam/α-Fe_2_O_3_NP nanocomposite (50 ppb).

The selectivity of a sensor is an important parameter for real aqueous sample applications. The selectivity of the electrode was investigated in the presence of possible interfering metal ions. The effects of different metallic ions, including Ag^+^, K^+^, Cd^2+^, Zn^2+^ and Fe^3+^, were tested for the possible interference with arsenic detection by the ZnO NRs/Ni-foam/α-Fe_2_O_3_NPs nanocomposite electrodes under similar experimental conditions. To study the influence of metal ions, metal salts like KCl, AgNO_3_, Cd(NO_3_)_2_·4H_2_O, Zn(NO_3_)_2_·6H_2_O and Fe(NO_3_)_3_·9H_2_O were employed. The concentration of the metal salts was maintained at 50 ppb in the 1 M KOH electrolyte solution. There was no strong interaction between arsenic(v) and the interfering species, as shown in Fig. 7. Therefore, these species do not interfere obviously with the electrochemical detection of arsenic(v). The results show that the fabricated sensor has good selectivity towards arsenic(v) ions.

### 3.6 Sensing mechanism of ZnO NRs/Ni-foam/α-Fe_2_O_3_NPs nanocomposite electrode

**Fig. 8:**
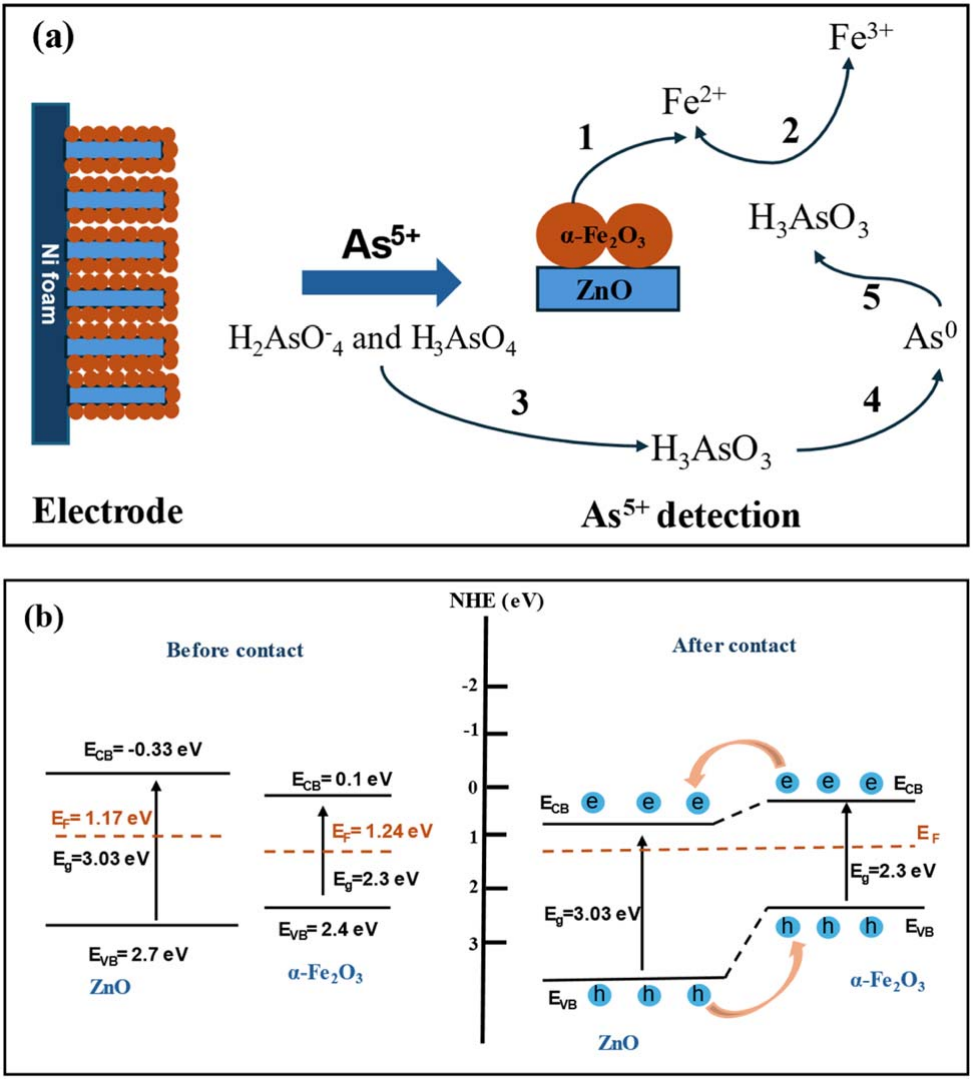
(a) Schematic of the sensing mechanism (concept based on ref. 66) and (b) equilibrium energy band structure diagram of the ZnO NRs/Ni-foam/α-Fe_2_O_3_NPs nanocomposite before and after contact between the ZnO NRs and α-Fe_2_O_3_NPs.

The mechanism of electrochemical detection of arsenic(v) by the ZnO NRs/Ni-foam/α-Fe_2_O_3_NPs nanocomposite electrode is presented in Fig. 8a. The nickel foam substrate facilitates the formation of ZnO NRs/α-Fe_2_O_3_NPs. The structure resembling a flower provides a large surface area and abundant active areas. This facilitates the fast flow of ions and electrons between the electrode and electrolyte. The enhanced performance of the ZnO NRs/Ni-foam/α-Fe_2_O_3_NPs nanocomposite electrochemical sensor compared with bare ZnO NRs/Ni-foam can be attributed to the distinctive electron transport and high absorption properties of the α-Fe_2_O_3_NPs in the nanocomposite towards arsenic(v) detection; arsenic(v) ions are adsorbed on the surface of the working electrode and interact with α-Fe_2_O_3_NPs during the electrochemical measurement, generating Fe^2+^ ions on the electrode surface due to electrochemical reduction.^67^ The arsenic(v) detection mechanism on the electrode surface begins with the adsorption of the H_2_AsO_4_^−^ and H_3_AsO_4_ species at the Fe^2+^ sites present on the electrode surface. Afterward, the arsenic(v) species are chemically reduced to H_3_AsO_3_ by the Fe^2+^ ions generated during the electrochemical measurement of the electrode. These reactions can be coupled with another electrochemical step, in which H_3_AsO_3_ is reduced to zero-valent arsenic on the electrode surface. Zero-valent arsenic is stripped during the anodic and cathodic scans. The reaction process is demonstrated below.^66^

(1) Reduction of Fe^3+^ to Fe^2+^ on the electrode surfaceα-Fe_2_O_3_ + 6H^+^ + 2e^−^ → 2Fe^2+^ + 3H_2_O

(2) Chemical reduction of As(v) to As(iii) on the electrode surface:H_2_AsO_4_^−^ + 3H^+^ + 2Fe^2+^ → H_3_AsO_3_ + 2Fe^3+^ + H_2_OH_3_AsO_4_ + 2H^+^ + 2Fe^2+^ → H_3_AsO_3_ + 2Fe^3+^ + H_2_O

(3) Electrochemical reduction of As(iii) to As (0) on the electrode surface:H_3_AsO_3_ + 3H^+^ + 3e^−^ → As^0^ + 3H_2_O

The mechanism of charge separation and transportation in the ZnO NRs/Ni-foam/α-Fe_2_O_3_NPs nanocomposite can be further explained by the generation of an n–n heterojunction between n-type ZnO and n-type α-Fe_2_O_3_ semiconductors, as shown in Fig. 8b. As the conduction band and valence band of α-Fe_2_O_3_ (0.1 eV and 2.4 eV) are more negative than those of ZnO (−0.33 eV and 2.7 eV), the electrons are transferred from the conduction band of α-Fe_2_O_3_ to ZnO. Meanwhile, the holes in the valence band of ZnO migrate to the valence band of α-Fe_2_O_3_. This migration and separation of electrons and holes are achieved at the heterojunction interface. When the n–n heterojunction of ZnO NRs/α-Fe_2_O_3_ is in contact with the electrolyte, electrons at the Fermi level align to attain equilibrium. The Fermi level in α-Fe_2_O_3_ is located in between its CB and VB at around 1.24 eV. The Fermi level of n-typical semiconductors generally lies at the bottom of the CB at around 0.1–0.2 eV.^68^ As a typical n-type semiconductor, the Fermi level of ZnO is at 1.17 eV. When the semiconductors ZnO and α-Fe_2_O_3_ are joined together, their Fermi levels overlap at 1.24 eV.

## 4 Conclusion

The fabrication of an α-Fe_2_O_3_NPs-modified ZnONRs/Ni-foam nanocomposite was successfully achieved *via* two separate growth steps using hydrothermal and dip-coating methods. The number of dip-coating cycles was varied to optimize electrode performance. The ZnO NRs/Ni-foam/α-Fe_2_O_3_NPs nanocomposite showed the best results when immersed 3 times in the precursor solution, demonstrating the highest oxidation current toward arsenic(v) detection compared with the samples prepared by immersing 2 and 4 times. The electrochemical performance of the optimal electrode in arsenic(v) detection was investigated in a wide range of arsenic concentrations from 0 to 50 μg L^−1^ by cyclic voltammetry. The results demonstrated LOD and LOQ values of 4.12 ppb and 13.74 ppb, respectively; the LOD is lower than the maximum permitted arsenic content recommended by the World Health Organization (WHO) in drinking water. Therefore, the ZnO NRs/Ni-foam/α-Fe_2_O_3_ NPs nanocomposite can be utilized as an electrode to develop efficient arsenic sensor systems for monitoring drinking water.

## Abbreviations

XRD: X-ray diffraction
XPS: X-ray photoelectron spectroscopy
FESEM: Field-emission scanning electron microscopy
ZnO: Zinc oxide
α-Fe_2_O_3_: Hematite
DI: Deionized
UV: Ultraviolet
NRs: Nanorods
NPs: Nanoparticles
CV: Cyclic voltammetry
LOD: Limit of detection
LOQ: Limit of quantification

## Data availability

All data relevant for the reproduction of the results presented in this work are included within the article.

## Author contributions

All authors have contributed to achieve this result, and they are aware and accept the submission.

## Conflicts of interest

The authors declare no conflict of interest.
